# Crowd behaviour during high-stress evacuations in an immersive virtual environment

**DOI:** 10.1098/rsif.2016.0414

**Published:** 2016-09

**Authors:** Mehdi Moussaïd, Mubbasir Kapadia, Tyler Thrash, Robert W. Sumner, Markus Gross, Dirk Helbing, Christoph Hölscher

**Affiliations:** 1 Center for Adaptive Rationality, Max Planck Institute for Human Development, Berlin, Germany; 2 Disney Research Zürich, Zürich, Switzerland; 3 Chair of Cognitive Science, ETH Zürich, Zürich, Switzerland; 4 Computer Graphics Laboratory, ETH Zürich, Zürich, Switzerland; 5 Computational Social Science, ETH Zürich, Zürich, Switzerland; 6 Computer Science Department, Rutgers University, NJ, USA

**Keywords:** collective behaviour, social systems, virtual laboratory, self-organization

## Abstract

Understanding the collective dynamics of crowd movements during stressful emergency situations is central to reducing the risk of deadly crowd disasters. Yet, their systematic experimental study remains a challenging open problem due to ethical and methodological constraints. In this paper, we demonstrate the viability of shared three-dimensional virtual environments as an experimental platform for conducting crowd experiments with real people. In particular, we show that crowds of real human subjects moving and interacting in an immersive three-dimensional virtual environment exhibit typical patterns of real crowds as observed in real-life crowded situations. These include the manifestation of social conventions and the emergence of self-organized patterns during egress scenarios. High-stress evacuation experiments conducted in this virtual environment reveal movements characterized by mass herding and dangerous overcrowding as they occur in crowd disasters. We describe the behavioural mechanisms at play under such extreme conditions and identify critical zones where overcrowding may occur. Furthermore, we show that herding spontaneously emerges from a density effect without the need to assume an increase of the individual tendency to imitate peers. Our experiments reveal the promise of immersive virtual environments as an ethical, cost-efficient, yet accurate platform for exploring crowd behaviour in high-risk situations with real human subjects.

## 1. Introduction

The dynamics of crowds implies major theoretical and real-world challenges [1–4]. As with many other de-centralized social and biological systems [5], the dynamics of crowd movements is driven by nonlinear amplification loops that promote the emergence of large-scale behavioural patterns. Recent progress in modelling and simulation techniques [6,7], coupled with advances in experimental methods [8,9] and live monitoring [3,10–12], has provided unprecedented amounts of theoretical and empirical insights into crowd movements, ranging from the emergence of ‘smart’ patterns of self-organization to their breakdown when deadly crowd disasters happen [13].

Despite these major advances, one important aspect of crowd behaviour that remains difficult to study is the collective dynamics that takes place under stressful emergency situations [4]. Empirical research has reported about several case studies of specific emergency evacuations, such as during the 9/11 attacks [14], the Love parade disaster [15], the Mecca pilgrimage [3,13] and other fire escape situations [16–18]. These works have highlighted prominent features of emergency escapes, such as the preference for familiar exit routes, the feeling of a common social identity within the crowd, and the nature of the fire alarm on people's reaction time. Other studies have demonstrated the contagious aspect of risk perception, suggesting that anxiety may spread from one pedestrian to another during stressful evacuations or that collective underestimation of the danger could lead to critical evacuation delays [19–22].

Yet, fine-grained data analyses are missing to extract the precise mechanisms driving collective behaviours during stressful evacuations. For example, it remains unclear to what extent pushing, overcrowding and peer imitation can affect the efficiency of egress. The main obstacle to answering these questions is the scarcity of detailed empirical data. Laboratory experiments are not suited for the study of emergency situations due to safety and ethical issues, and real-world observations similar to those described above are rare and difficult to evaluate. Consequently, most research in this domain is conducted by means of computer simulations based on simplified behavioural assumptions [2] or rely on analogies to animal models [23]. While computer simulations facilitate the collection of data in a controlled and cost-efficient way, the accuracy of the findings is inherently limited to the extent that the simulations mimic real crowds. Despite promising advances in this area [4,24], computer simulated agents cannot reliably emulate real human behaviour, especially for situations in which empirical data are difficult to obtain in the first place.

To overcome these limitations, we propose a novel approach to study the behaviour of large crowds of real experimental subjects, moving and interacting in shared immersive three-dimensional virtual environments [25,26]. In the last few years, an increasing number of studies have relied on virtual reality devices to investigate the behaviour of pedestrians, for example, by means of head-mounted displays or CAVE systems. Although some limitations were highlighted, such as a gender bias in handling the navigation controls [27], simple navigation tasks and route choice experiments were successfully conducted in virtual environments [28–30]. Virtual worlds have also been used to study features of emergency evacuations, but social interactions among pedestrians were absent [31] or limited to a single subject facing a group of simulated agents [32–35].

One crucial aspect of crowd dynamics lies in the social interactions that take place between individuals. These interactions create feedback loops and amplification effects and give rise to self-organized macroscopic patterns. It is therefore important to observe groups of participants moving and interacting simultaneously in the same environment. Notably, one study has managed to study crowd evacuation with groups of real people navigating simultaneously in the virtual world provided by the game Second Life [36], but the constraints imposed by the game structure made it difficult to keep a good control on all experimental variables.

This new technique is in concert with the recent development of computational methods in the social sciences that employ artificial environments to study the dynamics of large social systems [37], such as cultural markets [38], social networks [39] and collective problem-solving [40]. Here, we extend this experimental principle to crowd behaviour by allowing a large number of participants to navigate freely in an immersive virtual space and interact with one another in real time. This experimental technique enables the systematic study of crowd dynamics under extreme conditions, with complete control of experimental variables and without the prohibitive safety and ethical concerns of real-world experiments.

**Figure 1.**
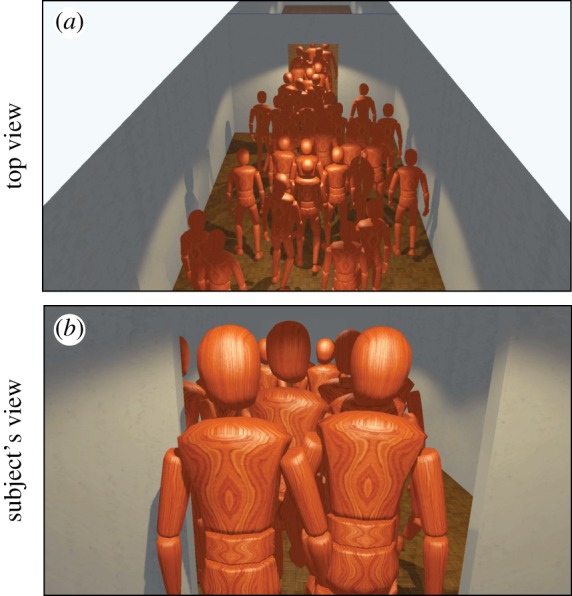
Illustration of the virtual environment. (*a*) Top-down view of a crowd of 36 participants passing through a bottleneck during a simple evacuation situation. Each pedestrian in this snapshot was controlled by a real experimental participant who can navigate freely in the environment. (*b*) First-person view of the same situation as seen by one participant located in the middle of the crowd.

In the present experiment, 36 experimental subjects participated simultaneously. Each participant sat in front of a computer screen and had a first-person view of the surrounding virtual environment, including the other participants (figure 1). Subjects navigated freely in the environment by using the computer mouse and the keyboard (see Methods; and electronic supplementary material, figure S1). We first assessed the validity of the method by replicating a series of previously conducted real-world crowd experiments using our virtual world platform (Studies 1 and 2). At both the micro and macro levels of observation, the virtual environment turns out to be a good proxy for real-life dynamics. Then, we explored the dynamics of high-stress evacuations in a series of experiments for which participants have to evacuate a building on fire under strong time pressure and heavy monetary penalization in case of failure (Study 3). We observed realistic panic movements and analysed emerging patterns of overcrowding and collective route choice. Our study demonstrates the promise of immersive multi-user virtual environments for the study of crowd dynamics, which opens a wide variety of research and applications.

## 2. Results

### 2.1. Method validation

**Figure 2.**
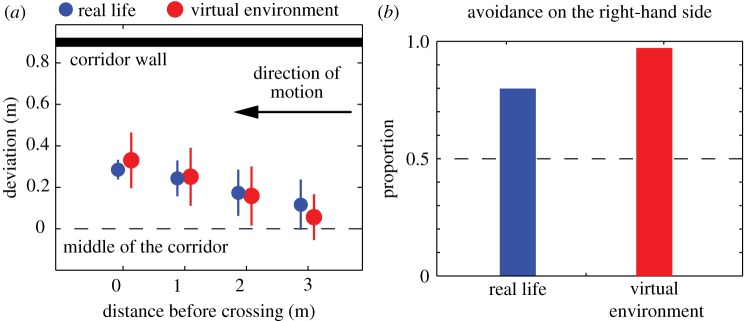
Comparison of virtual and real results during simple avoidance manoeuvres [41,42]. (*a*) Average lateral deviation of the walking trajectories during a simple avoidance task for which two participants moving in opposite directions avoid each other in a narrow corridor (real-life experiment in blue, *N* = 144; virtual environment in red, *N* = 561). Error bars indicate the standard deviation of the mean. (*b*) Proportion of participants avoiding each other on the right-hand sides during the same experiments.

In Study 1, we replicated a real-life experiment in which pairs of pedestrians are instructed to avoid each other in a narrow corridor [41] (electronic supplementary material, figure S3). The avoidance behaviours in the virtual environment conformed to real-life observations in terms of the shape of the trajectories and the choice of the passing side (figure 2*a*). Interestingly, we observed a marked side preference during avoidance manoeuvres in the virtual environment (figure 2*b*). In more than 95% of our replications, experimental subjects chose to avoid each other on the right-hand side. A two-proportion *Z*-test was used to compare the proportion of replications in which participants passed each other on the right side to a chance value of 50%, *Z* = 17.03, *p* < 0.001. This finding indicates that participants in the virtual corridor were following an existing social convention during avoidance manoeuvres. In the real-life experiment, 81% of the subjects avoided towards the right-hand side, but these proportions cannot be directly compared because the participants were drawn from different populations (i.e. in France for the real-life experiment and Switzerland for the virtual experiment). The main conclusion is that participants exhibited a marked side preference in the virtual corridor, suggesting that virtual worlds also capture some social aspects of pedestrian behaviour.

**Figure 3.**
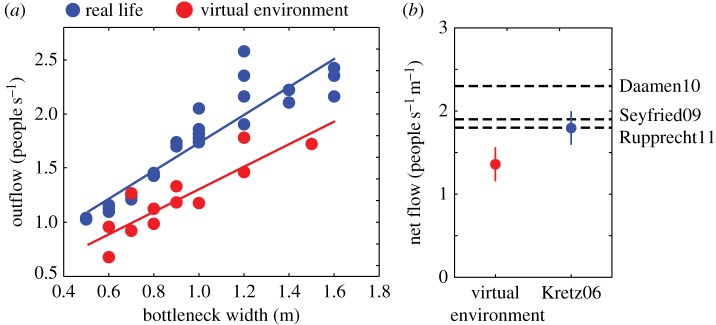
Flow through a bottleneck in real and virtual environment. (*a*) Flow of people through bottlenecks of varying width, measured during a group evacuation experiment in our virtual environment (red dots), replicating real-life experiments (blue dots). Lines of best fit are *f*(*x*) = 1.29*x* + 0.45 and *f*(*x*) = 1.04*x* + 0.27 for the real-life and virtual environments, respectively. (*b*) Net flow per unit of door width in the virtual environment (in red) and for study [42] (in blue). Error bars indicate the standard deviation of the mean. The three black dashed lines show the average values reported in three other real-life studies [9,43,44].

Study 2 tested the reliability of the virtual environment for reproducing *collective* crowd patterns. We studied 36 subjects performing a series of evacuation tasks, emulating the experimental design from [42]. Participants were immersed in a large virtual room and instructed to evacuate through a bottleneck of varying width, ranging from 60 cm to 150 cm (figure 1 and electronic supplementary material, figure S4). Consistent with real-life findings, the outflow of pedestrians increased linearly with the bottleneck width (figure 3). When compared with a larger body of real-life datasets, the outflow of participants seemed to be smaller in the virtual environment. This discrepancy can be due to a multitude of micro-navigation factors, such as differences in walking speed, acceleration or the shoulder movements between real and virtual environments. Although not identical, the observed trends in the virtual world are reasonably similar to the real-life dynamics to consider virtual environments as proxies for real-life dynamics.

### 2.2. Implementation of emergency evacuations

**Figure 4.**
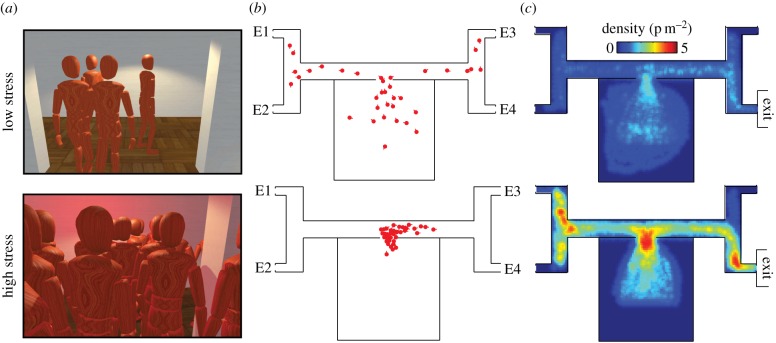
Comparison between low-stress and high-stress escape experiments. (*a*) Illustrative snapshots of the environment as seen by one participant approaching the decision zone in the low-stress (top) and high-stress (bottom) conditions. In addition to time pressure and the risk of losing money, the high-stress environment is characterized by stress-inducing factors such as lower luminosity and red blinking lights. (*b*) Representative top-down views of the participants' positions in both conditions. Under low stress, participants kept a certain distance from each other and tended to explore both branches of the main corridor. Under high stress, participants were densely packed and herded in the same branch. For each replication, the free exit door was randomly placed at one location among E_1_, E_2_, E_3_ and E_4_. In both examples illustrated here, the exit door was located at position E_2_. (*c*) Maximum density levels measured all over the environment, averaged across all replications (*N*_low-stress_ = 10 and *N*_high-stress_ = 12). Density levels hardly approach 2 persons m^−2^ in low-stress conditions, but reached up to 5 persons m^−2^ under high-stress—a very high value at which physical injuries might occur.

In Study 3, we performed a series of emergency egress experiments for low-stress (*C*_0_) and high-stress (*C*_1_) conditions (figure 4*a*). The environment consisted of a complex building with four possible exit locations E_1_, E_2,_ E_3_ and E_4_ through which participants were instructed to escape (figure 4*b* and electronic supplementary material, figure S5). For each replication, the functional exit door was placed at a randomly chosen exit location, whereas the other three exit locations are blocked. Participants were unaware of the location of the functional exit door, except for a certain proportion *k* of informed individuals who could see an arrow in the top of the screen that indicated the direction of the safe exit [45]. Participants knew that some group members may have been informed of the correct exit but cannot recognize them, thus mimicking the social uncertainty of real-life egress.

Stress is implemented by manipulating three experimental factors: (i) *time pressure*: participants had to escape the building within 50 s for *C*_1_. No time limit was imposed for *C*_0_. (ii) *Reward system*: throughout the experiment, participants could collect points that were converted into monetary bonuses at the end of the session (see Methods). In condition *C*_0_, participants were *rewarded* 50 points upon escaping the building. In condition *C*_1_, however, participants were *penalized* 100 points if they did not manage to escape in time, with no bonus for a successful escape. The reward system was therefore switched from the gain domain to the loss domain under high stress [46]. For both *C*_0_ and *C*_1_, participants were additionally penalized 1 point for colliding with another participant or obstacle. (iii) *Environmental factors*: a series of stress-inducing elements in the environment were implemented in *C*_1_ but not in *C*_0_. These elements included lower luminosity, red blinking lights and fires at the blocked exit locations.

### 2.3. Dynamics of emergency escape

We observed notable behavioural differences between the two conditions. In the absence of stress, participants tended to keep reasonably safe distances from their neighbours in order to avoid the collision penalty. Consequently, body contacts hardly occurred during low-stress evacuations (figure 4; electronic supplementary material, figure S6), as in similar real-life situations. By contrast, a high frequency of body contacts occurred in the high-stress condition, despite the application of the same collision penalty. Therefore, participants appeared ready to lose a considerable amount of points due to body collisions—and to impose the same penalty to their neighbours—to maximize the likelihood of escaping on time. On average, participants lost nearly the same amount of points due to body collisions (26 points per replication, s.d. = 13) and due to failures to escape (36 points per replication, s.d. = 38).

**Figure 5.**
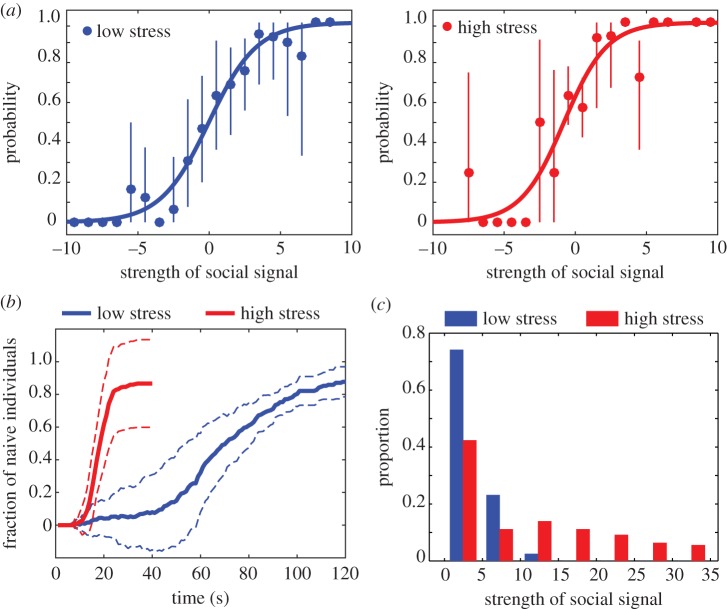
Herding dynamics. (*a*) Individual probabilities to choose the right-hand branch when arriving in the decision zone as a function of the social signal produced by the crowd at that moment. A positive signal indicates crowd movements directed towards the right side (a negative one, respectively, towards the left side). The left and right panels correspond to the low-stress and high-stress conditions, respectively. The response function was almost identical in both conditions, indicating that the observed herding patterns do not result from a change in the herding tendency but instead from the crowdedness. The fitted curves were obtained by minimization of the squared distance to the data points using the equation 1/(1 + e*^aS^*^+*b*^), resulting in *a* = −0.59 and *b* = 0.03 under no stress and *a* = −0.66 and *b* = 0.80 under high stress. (*b*) Average herding level *H*(*t*) indicating the fraction of uninformed individuals who chose the same branch as the majority of individuals, under low-stress (blue) and high-stress (red) conditions. The dashed lines represent the standard deviation of the average. Herding is stronger under high stress than under low stress (also illustrated in figure 4*b*), despite a similar individual response function shown in (*a*). (*c*) The distribution of the social signal strength shows that the social signal is weaker under low stress (blue) than under high stress (red).

The density levels also reflected relative crowdedness. It remained lower than 2 persons m^−2^ in *C*_0_, which is typically observed in everyday congested zones. Under high stress, however, the density level reached values up to 5 persons m^−2^, which violated all safety standards and was close to the critical threshold of crowd turbulence [13]. The most dangerous zones with the highest density levels were (i) areas in which a decision needed to be made, (ii) areas surrounding the exit where bottlenecks occurred and caused congestion, and (iii) dead ends where the flow of people returning after exploring a wrong option encountered the flow of those moving in the opposite direction (electronic supplementary material, figures S7 and S8). This overcrowding pattern was not only due to the reduction of interpersonal distance, but also due to the fact that most people *decided* to go in the same direction. We characterized the herding level *H*(*t*) at each time *t* by measuring *H*(*t*) = *p*_maj_(*t*) − *p*_min_(*t*), where *p*_maj_(*t*) represents the proportion of uninformed individuals who chose the branch where the majority of individuals converged at the end of the replication (analogously, *p*_min_(*t*) stands for the minority proportion). In *C*_0_, *p*_maj_ and *p*_min_ tended to increase at the same rate corresponding to an *H*-value close to 0 (figure 5*a*). After approximately 45 s, the flow of people who made an incorrect first decision reached the other branch, which was reflected by the subsequent gradual increase of *H*(*t*). In *C*_1_, however, the great majority of people chose the same branch at the beginning, and the herding level *H* approached 1 after a short time. In order to evaluate this pattern statistically, we fitted linear regression models to each trial and compared the slopes of the best fit lines for *C*_0_ to the slopes of the best fit lines for *C*_1_. As predicted, the slopes from *C*_1_ were significantly greater than the slopes from *C*_0_, *t*_14_ = 6.65, *p* < 0.001, *d* = 0.12, even after accounting for differences with respect to the length of each trial, *t*_14_ = 7.52, *p* < 0.001, *d* = 0.02. What are the behavioural mechanisms underlying the emergence of this herding pattern? We hypothesize that, under time and monetary pressure, subjects would increase their tendency to follow their neighbours as suggested in an early model of crowd panics [2], which would give rise to the observed herding pattern under high stress.

We tested this assumption by considering the response function *f*(*S*), which describes the individual probability to choose one branch or the other as a function of the social signal *S* produced by the crowd at the moment of a decision. In our experiment, the social signal *S*(*t*) is the movement of the crowd in the main corridor at the time of decision *t*, formally defined as
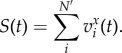
Here, *v*^*x*^_*i*_(*t*) is the horizontal component of participant *i*'s velocity indicating whether participant *i* was moving towards the right or the left side of the floor plan, and *N′* is the subset of participants who were present in the main horizontal corridor at time *t*. The empirically determined response function *f*(*S*) has a typical S-shape (figure 5*b*), indicating that individuals make use of social information when deciding where to go [47]. Surprisingly, however, the response functions measured under low-stress and high-stress conditions were quite similar, which was at odds with our first intuition. In order to evaluate this similarity statistically, we compared the correspondence between the response functions for *C*_0_ and *C*_1_ to the correspondence between randomly generated datasets. For values of *f*(*S*) that were missing for either *C*_0_ or *C*_1_, we randomly generated numbers between 0 and 1 from continuous, uniform distributions. One thousand replacement values were generated in this way for each missing value. For each set of original data with some proportion of randomly generated values, we then calculated the correlation between *C*_0_ and *C*_1_. We also randomly generated 1000 pairs of whole datasets and calculated the correlation between *C*_0_ and *C*_1_ in a similar way in order to produce a null distribution of correlation coefficients. An independent-samples *t*-test determined that the set of correlation coefficients derived from the original data was significantly greater than the correlation coefficients derived from random datasets, *t*_1998_ = 92.31, *p* < 0.001, *d* = 1.04. Note that, although this approach is relatively unsophisticated, it is also conservative compared with other approaches that replace missing values using the distribution of the original data (e.g. multiple imputation) [48].

Further analysis revealed that individuals were exposed to much stronger social signals under high-stress than low-stress situations due to increased local density levels, as shown by the distributions of |*S*| in figure 5*c*. While the values of were lower than 5 in 75% of the decisions made in low-stress conditions, the distribution was positively skewed under high-stress conditions and included values up to 35. Therefore, the *same* response function *f*(*S*) held in both conditions but applied to higher values of *S* under high-stress than under low-stress conditions. Put simply, pedestrians had a higher probability of following their neighbours when stress was high, simply because the neighbouring individuals were *more numerous* due to the increased density level. Herding, therefore, resulted from the crowdedness and not from a change in the individual tendency to imitate neighbours.

## 3. Discussion

The collective dynamics that takes place during stressful emergency evacuations is probably the least understood aspect of crowd behaviour, despite being crucial for crowd safety. In this work, we have proposed to observe crowds of *real* human subjects moving and interacting in *virtual* environments. Our approach offers important advantages and opens numerous research perspectives. First, it resolves safety and ethical issues and enables the systematic exploration of crowd behaviour under high-stress conditions with real human participants. Second, it is flexible and enables the exploration of crowd behaviours in potentially any virtual place without restrictions in terms of environment topology or size. Third, it allows for a rich variety of measured variables with high accuracy, including participants' field of view, and can be combined with eye-tracking or physiological measurement devices. Fourth, it permits the accurate control and manipulation of experimental variables such as light level, walking speed and body sizes.

We validated our experimental platform with respect to its ability to reliably replicate the dynamics of real crowds and demonstrate its potential to conduct previously infeasible studies such as the study of crowd behaviour in high-stress evacuations. However, our platform may benefit from further improvement. As the results of Study 2 suggest, the virtual environment will require further calibration work. While the bottleneck experiment in the virtual environment reproduces real-life data reasonably well, we have noted that the flow values were considerably lower in the virtual setting than in the real world. This difference could be due to a variety of differences at the micro-navigation level, such as dissimilarities in walking speeds or time delays when a keyboard key is pressed or released. Future work would therefore need to calibrate the control interface in order to produce more realistic crowd movements.

In the current state of development, organizing experiments with a larger number of participants (i.e. greater than the maximum capacity of the computer laboratory [36]) remains as difficult as for real-life experiments. In either case, experimental subjects need to be physically present in an experimental room, which involves other logistic challenges when the number of participants is large. As a consequence, between-group replications may be scarce. In our data, for example, we could not completely rule out group-specific biases (e.g. habituation effects), although none were detected (electronic supplementary material, figure S9). This issue could possibly be addressed by extending the experimental platform to a Web version for which participants would not need to be physically present in the laboratory [38,39,49]. Future work will therefore focus on extending our laboratory-based experimental approach to Web-based experiments, facilitating between-group replications and extending the number of simultaneous participants.

Our results leave open interesting questions that could be addressed in future studies. As past research has shown, social identification among individuals tends to promote inter-individual cooperation and enhance the efficiency of emergency evacuations [3,33]. The reward system that we have implemented and the separation of people into cubicles could have encouraged participants to behave in a more competitive manner, which could explain the observed number of collisions. In future works, social identification level could be manipulated experimentally in the virtual environment to address this issue.

According to current research on collective movements, interaction networks based on sensory information (e.g. vision) are crucial to understand emerging movement patterns [1,10,50]. One important direction of future work will therefore focus on establishing networks of visual contacts to determine precisely how visual cues propagate from person to person and how this information impacts herding behaviours [51]. This could be inferred from the position of individuals in the environment or by means of eye trackers.

In conclusion, the use of immersive multi-user virtual environments promises to be a powerful tool that can push the boundaries of crowd research in new and exciting directions, enabling new applications for urban planners and architects. Such applications include evaluating the quality of service and evacuation plans of building designs in virtual reality.

## 4. Methods

### 4.1. Experimental software

The experimental software was developed using the Unity3D game engine (Unity Technologies), ADAPT [52] for animating the virtual characters, and SmartFoxServer for the networking procedures. The platform immerses participants in a visually realistic virtual environment in which all users can freely navigate and see the other participants in real time. Subjects had a first-person view of the environment and could navigate by means of a keyboard and mouse. Navigation included three degrees of freedom: forward/backward translations, left/right translations and left/right rotations (electronic supplementary material, figure S1 and video S1). The control interface was tested in a previous study [53] and yielded the best navigation performance when compared with two other control solutions (keyboard-only and joystick) with respect to real human walking trajectories. For simplicity, we assume homogeneous virtual characters (height: 1.8 m; shoulder width: 0.25 m; maximum forward walking speed: 1.3 m s^−1^; backwards and lateral moving speed: 0.6 m s^−1^). A circular collision check with a diameter equal to the shoulder width was implemented to ensure that virtual pedestrians do not overlap in crowded situations.

### 4.2. Experimental design

Two experimental sessions took place in June and December 2014. For each session, 36 experimental subjects were hired and invited to the laboratory. They received between 20 and 50 CHF for their participation depending on performance. Data were collected in the ETH Decision Science Laboratory (DeSciL) which independently approved the experimental procedures according to its human subjects regulations. Informed consent was obtained from all participants based on DeSciL requirements. Participants were seated in a room containing 36 cubicles, each containing a desktop computer. They could not see the screen of the other participants and were not allowed to communicate with each other during the experiment. Subjects were instructed to wear headphones for the duration of the experiment. Each experimental session started with a training phase of approximately 40 min, during which all participants learned how to navigate in the virtual environment. In this phase, subjects had to complete a step-by-step tutorial designed to review all possible movements (electronic supplementary material, figure S2). During the first experimental session, we conducted Studies 1 and 2, while Study 3 was conducted during the second session. Throughout each experiment, subjects earned points that were converted to monetary compensation at the end of the session. In all experiments, participants were penalized 1 point every time they collided with another participant or obstacle. Participants initially started with 1000 points in the second session to compensate for the expected losses from the high-stress experiment.

*Study 1* is the replication of a real-life experiment conducted previously [41] in which pairs of participants moving in opposite directions had to avoid each other in a narrow corridor. The 36 subjects were randomly grouped in pairs and placed at each end of a straight corridor (length = 8 m; width = 1.8 m; electronic supplementary material, figure S3). Each participant was instructed to reach the other end of the corridor without colliding. Any collision with the corridor walls or the other participant resulted in a penalty of 1 point. At the end of each replication, new pairs of participants were randomly assigned. The 18 pairs of subjects performed the experiment simultaneously in 18 independent virtual corridors. We collected 561 replications of this experimental condition in approximately 10 min, which illustrates the flexibility of our experimental platform.

*Study 2* is a replication of a real-life evacuation experiment conducted previously [42]. Participants were initially located in a large room (width = 10 m; length = 4 m) and instructed to walk through a bottleneck after the starting signal to a finish line located 10 m after the bottleneck (electronic supplementary material, figure S4). The bottleneck width varied from 0.6 m to 1.5 m. We performed 14 replications in total, two for each bottleneck width. Two replications were later discarded because some participants deliberately blocked the outflow by standing in front of the bottleneck door. Participants received a bonus of 100 points after reaching the finish line and had no incentives for completing the task faster than others. The different bottleneck widths appeared in a random order.

*Study 3* was divided into a first block of 10 replications for the low-stress condition and a second block of 12 replications for the high-stress condition. Participants did not see the map of the environment, but they were allowed to explore it freely during a preliminary training session. In the low-stress condition, subjects were instructed to find the exit door of a complex building (electronic supplementary material, figure S5). No time limit was imposed to find the exit door, and subjects were awarded 50 points at the end of each replication. The high-stress condition was the same except for the three following stress-inducing factors. (i) A time limit of 50 s was imposed. The time limit was calibrated such that participants had enough time to explore one exit but not enough to explore a second one if the first option was not correct. (ii) Subjects who did not manage to escape within the time limit received a penalty of 100 points. Those who were successful did not receive any additional bonus. (iii) A set of stress-inducing elements were added to the environment including red blinking lights, lower luminosity, fire blocking the wrong exit doors and the sounds of an alarm. In each replication of the low- and high-stress conditions, a certain proportion *k* of subjects were informed about the location of the exit. Informed participants could see an arrow on the top of their screen pointing towards the exit. All subjects knew that some of them could be informed but did not know how many and could not recognize informed individuals. We varied the proportion of informed subjects in *k*: 0%, 10%, 33% and 100%. The purpose of this manipulation was to give participants the feeling that some of their neighbours might know the location of the exit, which mimics the uncertainty of real-life evacuations. The proportion of informed individuals *k*, as well as the location of the exit door were randomized between trials.

## Supplementary Material

Supplementary Materials

## Data accessibility

The datasets supporting this article are available at http://mehdimoussaid.com/Files/dataMoussaid2016.zip.
