# Supplementary material for: Crowd behaviour during high-stress evacuations in an immersive virtual environment
Source: J R Soc Interface. 2016 Sep;13(122):20160414. doi: 10.1098/rsif.2016.0414 (PMC5046946; doi:10.1098/rsif.2016.0414)
Supplement: Supplementary Materials [file rsif20160414supp1.doc]

# Crowd Behaviour during High-Stress Evacuations in an Immersive Virtual Environment

###

# **Supplementary Materials**

**
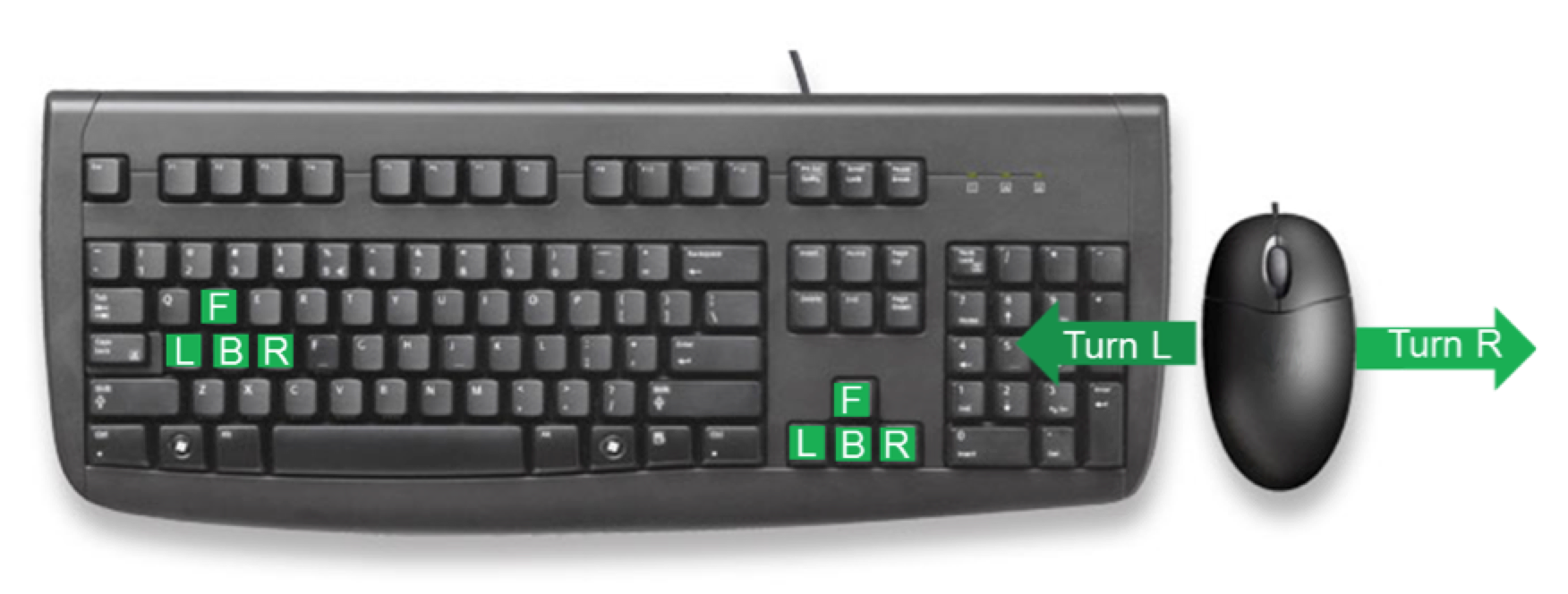
**

**Figure S1**: Control interface. Participants can navigate in the virtual environment by means of the keyboard and the mouse. Horizontal movements of the mouse are used to control the left and right rotation of the character (vertical movements have no effect). In addition, participants can use four keyboard keys that are highlighted in green: ‘*F*’ for forward movement, ‘*B*’ for backward movements, ‘*L*’ for left translation, and ‘*R*’ for right translation. Subjects can indifferently use one of the two possible sets of keys (one located on the left of the keyboard, and one located one the directional arrows), depending on their personal preferences. This control setup generated the best navigation performance as compared to two other control solutions (see Materials and Methods).

**Figure S2**: Overview of the environment used for the step-by-step tutorial. Participants started at the location of the blue point, facing the direction indicated by the blue arrow. They were alone in the virtual room, and had to perform the following sequence of navigation tasks: (*1*) use the mouse to look in the direction of the green flag on the right, (*2*) use the mouse to look in the direction of the red flag on the left, (*3*) use the mouse to move back in the initial direction, (*4*) use the ‘F’ key to move forward and touch the grey wall, (*5*) use sequentially the ’*R*’,’*F*’, and ‘*L*’ keys to bypass the grey wall, (*6*) pass the door and enter the next room. Once the subject enters the next room, a flag appeared at position P_1_. Participants were instructed to use all available controls to reach that flag. Then, the flag at position P_1_ disappeared and a new flag appeared at position P_2_ that the subject had to reach again, and so on until the last flag at position P_6_ was reached.

**Figure S3**: Illustration of the experiment E1. (A) The overview of the environment used for the avoidance experiment is the same as for a real-life experiment that was conducted in the past [(33)](https://paperpile.com/c/3Ac6tv/sakOa). Participants were initially located at both ends of a narrow corridor and facing each other (as indicated by the blue and red circles). At the starting signal, they were instructed to reach the end of the corridor and pass the finish line, without colliding with the other subject. (B) First-person view of one subject during one replication of the experiment.

**Figure S4**: Overview of the environment used for experiment E2. All the 36 subjects participated in this experiment simultaneously. Participants were initially located in the left room. Each subject was randomly positioned on one of the 36 possible starting positions indicated by the blue circles. Subjects were instructed to pass the bottleneck and reach the finish line located 10 meters ahead. No incentive was given to finish faster than other participants. Collisions with walls or other subjects were penalized by the loss of one point. The width of the bottleneck varied between 0.6m and 1.5m across repetitions. This design is a replication of a real-life experiment conducted in the past [(14)](https://paperpile.com/c/3Ac6tv/jpf7y).

**Figure S5**: Overview of the environment used in experiment E3. All the 36 subjects participated in this experiment simultaneously, and were initially located in the grey zone. Subjects were instructed to find the exit door and enter the safe zone. They knew that the free exit door was randomly located in one of the four possible locations E_1_, E_2_, E_3_ and E_4_, but didn’t know which one exactly. The three other door locations were blocked. The starting room leads to a decision zone where subjects could choose to go either left or right. The analyses that are presented in **Figure 5** focus on the choice of participants approaching this decision zone. Each branch leads to a second junction such that participants could not see the exit door directly from the first decision zone and had to decide solely based on what they saw in the main horizontal corridor.

**Figure S6**: Physical interactions during low-stress (C_0_) and high-stress (C_1_) conditions for E3. (A) Distribution of the number of collisions experienced by each individual in all replications under low-stress (blue bars) and high-stress (red bars) conditions. Body contacts hardly occurred during low-stress evacuations, which is comparable to most real-life situations, whereas a high frequency of unintentional body contacts and pushing occurred under high-stress. (B) Total amount of collisions per replication under low-stress (blue) and high-stress (red) conditions. The severity of overcrowding seems to depend on the number of informed individuals in the crowd under high stress, but this tendency cannot be statistically confirmed due to the small sample size.

**Figure S7**: Average density levels under low stress (top) and high stress (bottom) conditions for E3. The heat maps represent the average density levels measured in each settings, averaged across all replications. For this figure, the maps were normalized such that the exit door is always located on the right-hand side of the environment. The maps on the left show the average density values for all situations where the exit was located on the upper branch, whereas the maps on the right represent situations where the exit is on the lower branch.

**Figure S8**: Maximum density levels under low stress (top) and high stress (bottom) conditions for E3. The heat maps represent the *maximum* density levels measured in each settings, averaged across all replications. The same normalization procedure as in figure S7 was applied.

**Figure S9:** Control for habituation effects for E3. (A) Total number of collisions between individuals, (B) Number of people who escaped from the building after 50 seconds, and (C) Maximum observed density levels at time t=30s (red) and t=50s (blue), for all replications. Replications are represented in the order where they were conducted. A qualitative shift is visible for all measures when the switch from condition C_0_ (no stress) to condition C_1_ (high stress) occurs. The transition from C_0_ to C_1_ is marked by the black dashed line between replication 10 and 11. The trends are not gradually changing within each condition, indicating the absence of significant habituation effects.
